# Development and validation of an exome-based SNP marker set for identification of the St, J^r^ and J^vs^ genomes of *Thinopyrym intermedium* in a wheat background

**DOI:** 10.1007/s00122-019-03300-9

**Published:** 2019-02-14

**Authors:** Andras Cseh, Caiyun Yang, Stella Hubbart-Edwards, Duncan Scholefield, Stephen S. Ashling, Amanda J. Burridge, Paul A. Wilkinson, Ian P. King, Julie King, Surbhi Grewal

**Affiliations:** 1 Nottingham BBSRC Wheat Research Centre, Division of Plant and Crop Sciences, School of Biosciences, The University of Nottingham, Sutton Bonington Campus, Loughborough, UK; 2 Present Address: Molecular Breeding Department, Agricultural Institute, Centre for Agricultural Research, Hungarian Academy of Sciences, Martonvásár, Hungary; 3 Life Sciences, University of Bristol, Bristol, UK

## Abstract

***Key message*:**

**Cytogenetic analysis and array-based SNP genotyping of wheat–** ***Th. intermedium*** **introgression lines allowed identification of 634 chromosome-specific SNP markers across all twenty-one chromosomes of** ***Th. intermedium*** **(StJ** ^**r**^ **J** ^**vs**^ **, 2** ***n*** **= 6** ***x*** **= 42).**

**Abstract:**

*Thinopyrum intermedium* (2*n* = 6*x* = 42, StJ^r^J^vs^) is one of the most promising reservoirs of useful genes including tolerance to abiotic stresses, perenniality and disease resistance not available in the cultivated bread wheat. The transfer of genetic diversity from wild species to wheat offers valuable responses to the effects of climate change. The new array-based single-nucleotide polymorphism (SNP) marker technology provides cheap and easy-to-use molecular markers for marker-assisted selection (MAS) in wheat breeding programmes. Here, we focus on the generation of a new chromosome-specific SNP marker set that can be used to characterize and identify the *Th. intermedium* chromosomes or chromosome segments transferred into wheat. A progressive investigation of marker development was conducted using 187 various newly developed wheat–*Th. intermedium* introgression lines and the Axiom^®^ Wheat-Relative Genotyping array. We employed molecular cytogenetic techniques to clarify the genome constitution of the *Th. intermedium* parental lines and validated 634 chromosome-specific SNPs. Our data confirmed the allohexaploid nature of *Th. intermedium* and demonstrated that the St genome-specific GISH signal and markers are present at the centromeric regions of chromosomes 1J^vs^, 2J^vs^, 3J^vs^ and 7J^vs^. The SNP markers presented here will be introduced into current wheat improvement programmes, offering a significant speed-up in wheat breeding and making it possible to deal with the transfer of the full genetic potential of *Th. intermedium* into wheat.

**Electronic supplementary material:**

The online version of this article (10.1007/s00122-019-03300-9) contains supplementary material, which is available to authorized users.

## Introduction

The domestication of wheat (*Triticum aestivum* L., BBAADD, 2*n* = 6*x* = 42) and 10,000 years of wheat breeding practices have led to a genetic bottleneck. In the light of current climate models, there is an immediate demand to increase genetic diversity of cultivated wheat to ensure stress adaptability (biotic or abiotic) and food security in future. Wild relatives of wheat provide the opportunity to introduce novel genes via wide hybridization and thus represent a valuable source of genetic variation (Zhang et al. 2017). The perennial intermediate wheatgrass (*Thinopyrum intermedium* Barkworth & D.R. Dewey, StJ^r^J^vs^, 2*n* = 6*x* = 42) is one of the most promising gene reservoirs within the Triticeae family. It is frequently used in modern pre-breeding programmes as a donor of drought tolerance, high-temperature tolerance and salinity tolerance genes and is considered as a useful genetic material providing resistance against a wide spectrum of fungal pathogens (wheat leaf rust, stripe rust, stem rust, powdery mildew and eyespot; immunity to smut, leaf blight, root rot) and barley yellow dwarf virus and stripe mosaic viruses (Friebe et al. 1996; Li et al. 2005, 2012; Li and Wang 2009; Zeng et al. 2013; Danilova et al. 2017). Additionally, *Th. intermedium* may have the potential to improve wheat end-product quality and to provide perennial growth habit (Li et al. 2013).

Two main complications hindered the effective deployment of introgressed genes from *Thinopyrum* species into wheat: (1) in most of the cases, the F_1_ hybrid endosperm is shrivelled and thus seeds did not germinate under normal conditions; (2) the evaluation of recombinant lines was generally performed manually through intensive and time-consuming cytogenetic methods and could not be analysed by high-throughput techniques (Friebe et al. 1991; Lukaszewski et al. 2005). The first difficulty can be addressed by embryo rescue and tissue culture techniques, which allow a greater access to genetic resources (Sharma and Gill 1983). The second challenge presented by the detection of introgressions requires the development of high-throughput chromosome-specific molecular markers covering the entire wild relative genome.

While a range of molecular markers specific to the *Th. intermedium* genome have already been reported, such as simple sequence repeats (SSR) (Ayala-Navarrete et al. 2010), expressed sequence tags (EST) sequences (Wang et al. 2010; Danilova et al. 2017), PCR-based landmark unique gene (PLUG) markers (Hu et al. 2014; Zhan et al. 2015) and specific-locus amplified fragment sequencing (SLAF) markers (Li et al. 2016), their number is still limited and does not cover the whole *Thinopyrum* genome. Single-nucleotide polymorphism (SNP) markers have been developed by exploiting recent advances in next-generation sequencing platforms to provide cheap and easy-to-use molecular markers for marker-assisted selection in breeding programmes. Gene-associated SNP-based identification of the intermediate wheatgrass chromosomes in the background of wheat is challenged by the hexaploid nature and complex genome composition of both species and the high degree of similarity between the homoeologous groups. Additionally, wheat chromosome constitution is well known, but the *Th. intermedium* genetic make-up is still being unravelled.

*Th. intermedium* is an allohexaploid species having three genomes with chromosomes sorted in seven homoeologous groups. *Th. intermedium* is proposed to be formed by an ancient hybridization event between the diploid *Pseudoroegneria strigosa* (2*n* = 2*x* = 14, StSt) and a segmental tetraploid carrying J^r^ and J^vs^ genomes (Wang et al. 2015). J^r^ and J^vs^ genomes represent ancestral genomes of present-day J^b^ of *Th. bessarabicum* and J^e^ of *Th. elongatum*, respectively (Wang et al. 2015). J^vs^ is distinct from J^b^ as it retained repetitive sequences from the V genome (*Dasypyrum villosum* (L.) P. Candargy (genome VV, 2*n* = 14), while J^r^ carries a long terminal repeat (LTR) originating from the R (*Secale cereale*) genome (Kishii et al. 2005; Mahelka et al. 2011; Wang et al. 2015). The St genome in intermediate wheatgrass is highly similar to the present-day St of *Pseudoroegneria,* and chloroplast sequence data indicate that *Pseudoroegneria* is the most likely maternal progenitor of *Th. intermedium* (Liu and Wang 1993; Mahelka et al. 2011).

By using genomic in situ hybridization (GISH), genomes of *Th. intermedium* can be visualized and thus any introgression into the wheat background can be clearly confirmed (Han et al. 2003). This methodology, although labour-intensive for routine analysis of breeding material, is useful for validation during marker development. An exome-based SNP array (Axiom^®^ HD Wheat-Relative Genotyping Array) and cluster identification algorithms have been recently developed to infer detailed detection of introgressions into wheat from its wild relatives in a cost-effective manner (King et al. 2017).

The present study focused on the development of a large number of wheat/*Th. intermedium* introgression lines by using the ‘shotgun introgression’ approach aiming to transfer the full genetic potential of *Th. intermedium* into wheat. We used the Axiom^®^ Wheat-Relative Genotyping Array to detect the introgressions and validated the results by molecular cytogenetic methods. We present a new SNP marker set for identification of *Th. intermedium* chromosomes in a wheat background, while the new wheat/*Th. intermedium* introgression lines can be used as a valuable genetic tool in future wheat improvement programmes.

## Materials and methods

### Plant material

Two accessions of hexaploid *Th. intermedium* (accessions 401141 and 440016 obtained from Germplasm Resource Unit (GRU) at John Innes Centre (JIC), UK) were used to produce F_1_ wheat/wheatgrass interspecific hybrids. In order to improve the recombination event between the chromosomes of a wild relative and wheat, we produced the F_1_ lines by using a *ph1* mutant line of wheat cv. ‘Paragon’. The hybrids were backcrossed with the normal wheat parent (*Ph1/Ph1*) to generate BC_1_ populations. The BC_1_ individuals and their resulting progenies were then recurrently pollinated with ‘Paragon’ wheat to produce BC_2_, BC_3_ and BC_4_ populations (Table S1).

For GISH, accessions of *Pseudoroegneria strigosa* ssp. *Aegilopoides* (2*n* = 2*x* = 14 StSt; PI 531754), *Thinopyrum bessarabicum* (2*n* = 2*x* = 14 J^b^J^b^; PI 531710) and *Dasypyrum villosum* (2*n* = 2*x* = 14 VV; PI 639751) were obtained from Germplasm Resources Information Network of the US Department of Agriculture (USDA).

### In situ hybridization

The protocol for chromosome preparations from root tips was as described by Kato et al. (2004) and King et al. (2017). Genomic DNA was isolated using a CTAB method (Zhang et al. 2013) from young leaves of the three putative diploid progenitors *P. strigosa* (St genome), *Th. bessarabicum* (J^b^ genome) and *D. villosum* (V genome). The genomic DNA o*f Th. bessarabicum* and *D. villosum* was labelled by nick translation with biotin-14-dATP (BioNick Labeling System; Invitrogen, USA), and the genomic DNA of *P. strigosa* was labelled with digoxigenin-11-dUTP (DIG-Nick Translation Mix; Roche Diagnostics). Only two probes were used in each GISH experiment: either *Th. bessarabicum* labelled with biotin-14-dATP (green) together with the genomic DNA of *P. strigosa* labelled with digoxigenin-11-dUTP (red) or *D. villosum* labelled with biotin-14-dATP (light blue) together with genomic DNA of *P. strigosa* labelled with digoxigenin-11-dUTP (red). GISH was carried out according to Molnár-Láng et al. (2000) with minor modifications (Sepsi et al. 2008). Unlabelled wheat (Paragon) genomic DNA was used as blocking DNA at a ratio of 40:1. Biotin and digoxigenin signals were detected using streptavidin–FITC (Roche) and anti-digoxigenin–rhodamine Fab fragments (Roche), respectively. The slides were mounted in Vectashield antifade solution (Vector Laboratories) containing 2 μg/mL 4′-6-diamino-2-phenylindole (DAPI). After rinsing off the GISH hybridization signals in 4 × SSC Tween at 25 °C for 2 h, the multicolour FISH was carried out using the directly labelled Afa family and pSc119.2-1 probes. Afa family DNA sequence was amplified by PCR and labelled by nick translation with Alexa Fluor^®^ 594-5-dUTP (Invitrogen; C11400) (Nagaki et al. 1995). The pSc119.2-1 synthetic oligonucleotide was 5′ end-labelled with Alexa Fluor^®^ 488 (Tang et al. 2014). All slides were analysed using a Leica DM5500B epifluorescence microscope (Leica Microsystems, Wetzlar, Germany) with separate filters for detecting DAPI (blue), Alexa Fluor 488 (green) and Alexa Fluor 594 (red). Photographs were taken using a Leica DFC350 FX digital camera, and the images were analysed with Isis software (MetaSystems, Altlussheim, Germany).

### Genotyping via an Affymetrix SNP array

The Axiom^®^ 35 K Wheat-Relative Genotyping Array was used to genotype 206 samples using the Affymetrix GeneTitan^®^ system, and allele calling was carried out using the procedure described by King et al. (2017). The SNPs were classified by SNPolisher R package using SNP performance metrics. These categories were as follows: (1) ‘Poly High Resolution’ (PHR), which were codominant and polymorphic, with at least two examples of the minor allele; (2) ‘No Minor Homozygote’ (NMH), which were polymorphic and dominant, with two clusters observed; (3) ‘Off-Target Variant’ (OTV), which had four clusters, one representing a null allele; (4) ‘Mono High Resolution’ (MHR), which were monomorphic; (v) ‘Call Rate Below Threshold’ (CRBT), where SNP call rate was below threshold but other cluster properties were above threshold; and (6) ‘Other’, where one or more cluster properties were below threshold. For selection of chromosome-specific SNPs, the PHR and selected CRBT SNPs were used as they provided good cluster resolution where each SNP essentially behaves like a diploid. CRBT markers with > 6% missing data were removed prior to analysis.

### Selection of *Th. intermedium* chromosome-specific SNPs

Individuals from backcross populations of wheat–*Th. intermedium* hybrids were genotyped with the Axiom^®^ Wheat-Relative Genotyping Array. Along with triplicates of the parental lines, Paragon and both *Th. intermedium* accessions, 197 lines comprising F_1_, BC_1_, BC_2_, BC_2_F_1_, BC_3_, BC_3_F_1_ and BC_4_ populations of the wheat–*Th. intermedium* hybrids were genotyped altogether, making a total of 206 lines. SNP markers showing (1) heterozygous calls for either parents, (2) no polymorphism between the wheat and *Th. intermedium* parents and/or (3) no calls for either parents were removed using Flapjack™ (Milne et al. 2010). The resulting markers were sorted into linkage groups in JoinMap^®^ 4.1 (van Ooijen 2011) with a LOD score of 40 and a recombination frequency threshold of 0.1 using the Haldane mapping function (Haldane 1919). All markers that did not show any heterozygous call or were unlinked were ignored, and only the highest-ranking linkage groups with more than 25 markers were selected. These were exported and assigned to chromosomes using information from the Axiom^®^ Wheat HD Genotyping Array (Winfield et al. 2016). Erroneous markers showing a unique pattern of segregation that was either not observed in the previous backcross generation or not consistent with the recombination of neighbouring markers in the group, in different samples, were also removed. The marker order within each linkage group for the St and the J^r^ genomes was determined through cytogenetic analysis of the genome constitution and organization along with BLAST analysis against the wheat reference sequence (RefSeq v1.0; International Wheat Genome Sequencing Consortium et al. 2018) as described in the next section. The linkage groups were then used to form a physical SNP map of the three genomes of *Th. intermedium*. McGISH results were used to select lines carrying only 1–3 *Th. intermedium* chromosomes (BC_2_ and BC_3_ lines) in order to identify and validate St and J^r^ chromosome-specific linkage groups in the physical map. Lines with J^vs^ chromosomes showed both the St- and J^r^-specific markers of the same homoeologous group.

### Identification and characterization of recombinant wheat–*Th. intermedium* lines

When identifying translocations with the help of the physical map, three different segment sizes were expected: short terminal segment, telosome and larger than a telosome. Mapping data revealing a short wild relative telomeric chromosome segment suggested the presence of a terminal translocation and was, thus, used as a basis for subsequent GISH/FISH analysis. GISH, then, confirmed the formation of a telomeric translocation, and subsequently, FISH helped to identify the wheat chromosome involved in the translocation. If the map indicated the addition of a telosome, GISH determined if it is a telosomic addition or a centric fusion, while FISH revealed the identity of the wheat chromosome arm involved in the Robertsonian translocation. If the chromosome segment detected by the map was larger than a telosome, GISH visualized the translocation segment, while FISH indicated the origin of the wheat chromosome involved in the translocation.

### Comparative analysis

Synteny analysis was carried out using sequence information of the markers located on individual *Th. intermedium* chromosomes. Sequences of the markers within a linkage group were used in a BLAST analysis (e value cut-off of 1e-05) against the wheat reference genome (RefSeq v1.0; International Wheat Genome Sequencing Consortium et al. 2018), and the best BLAST hit (BBH), providing the physical position of the markers, against each of the three genomes of wheat was obtained, where available. To generate Fig. 6, we selected marker groups (1) unique to St and J^r^ genomes, (2) markers present on both the J^r^ and J^vs^ chromosomes, (3) markers present on the St and J^vs^ chromosomes, (4) markers present on both J^r^ and St chromosomes and (5) markers present on all 3 *Th. intermedium* genomes and plotted them against their physical positions on the D genome of wheat. Circos plots were visualized using Circos v. 0.67 (Krzywinski 2009) to observe synteny between *Th. intermedium* genomes and the wheat D genome.

## Results

### Chromosome constitution of *Th. intermedium* parental lines

**Fig. 1:**
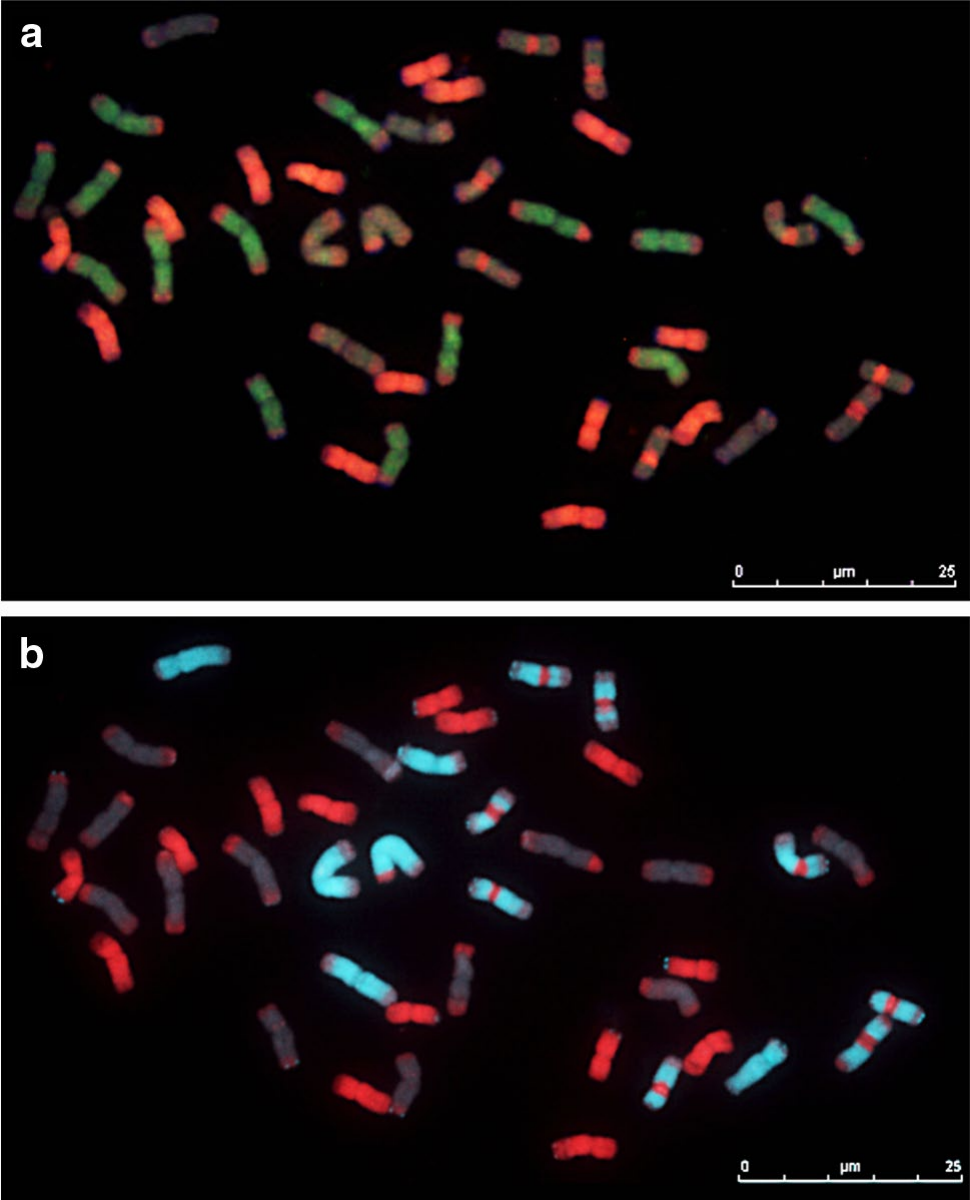
Sequential multicolour GISH analysis of *Thinopyrum intermedium* (StStJ^r^J^r^J^vs^J^vs^, 2*n* = 6*x* = 42). **a***Thinopyrum bessarabicum* (J^b^J^b^) probe (green) detected 14 J^r^ genome chromosomes, while *Pseudoroegneria spicata* (StSt) probe (red) showed 14 St genome chromosomes. **b***Pseudoroegneria spicata* (red) probe labelled the same 14 chromosomes as seen on (**a**) while *Dasypyrum villosum* probe (VV) (light blue) detected 14 J^vs^ chromosomes

Chromosome constitution of two accessions of *Th. intermedium* (accessions: 401141, 440016) were analysed by means of multicolour genomic in situ hybridization (mcGISH). Genomic DNAs of *Pseudoroegneria strigosa subsp. aegilopoides* (StSt) and *Thinopyrum bessarabicum* (J^b^J^b^) were applied as probes to metaphase chromosome preparations of the *Th. intermedium* accessions. Subsequently, the hybridization signal was washed down and the slides were reprobed with labelled *Dasypyrum villosum* (VV) and *Pseudoroegneria strigosa subsp. aegilopoides* (StSt) DNAs. The results suggest that *Th. intermedium* had 42 chromosomes originating from three different genomes (Fig. 1a–b). *P. strigosa* (red), *Th. bessarabicum* (green) and *D. villosum* (light blue) produced characteristic signals on separate chromosome sets, presumably representing the three distinct subgenomes (St, J^r^ and J^vs^) of *Th. intermedium*. The wheatgrass accessions used in this study showed 14 St genome chromosomes strongly labelled as red by the St genomic DNA probe (Fig. 1a–b). The J^b^ genomic probe revealed 14 J^r^ chromosomes, labelled as green, that showed red St genome signal at the subtelomeric regions (Fig. 1a). The J^vs^ genome proved to have the most complex chromosome constitution as chromosomes were painted by V genomic DNA probe in light blue, while the centromeric region of eight chromosomes showed strong St genome signal in red (Fig. 1b). In addition, the St genomic probe produced a dispersed red signal over the telomeric regions of all 14 J^vs^ chromosomes.

### Generation of introgressions

From the F_1_ to BC_4_ generations of the crossing programme, 768 ears were pollinated resulting in 3282 seeds, of which 197 plants were genotyped (Table S1). The lowest germination rate was observed within the F_1_ generation (31.7%). Nevertheless, average germination for the whole programme reached 66%. F_1_ hybrids showed the lowest fertility: only 30.5% of the ears produced seeds, while 62.8%, 67.5% and 98.5% of the BC_1_, BC_2_ and BC_3_ ears were fertile, respectively. The average number of seed set per crossed ear increased from generation to generation from 0.4 in the F_1_ to 12.9 in the BC_3_ plants. Self-fertility of the three backcross generations was higher compared to the F_1_ generation. Twenty-nine of the 33 germinated F_1_ seeds reached maturity and set seed when pollinated with wheat (Table S1); therefore, *Th. intermedium*/wheat introgressions developed in the present programme originated from these 29 individuals and their progenies. Thirty-three of the germinated 46 BC_1_ seeds reached maturity and set seed when pollinated with ‘Paragon’. In total, 687 BC_2_ seeds were obtained from 239 crossed BC_1_ ears. Following random selection, 101 BC_2_ seeds were germinated of which 74 plants survived. These were subsequently pollinated with ‘Paragon’ resulting in 787 BC_3_ seeds. From the BC_3_ generation, 75 seeds were selected at random and crossed to ‘Paragon’ yielding in 1757 BC_4_ seeds (Table S1). McGISH was used to screen the BC_2_ and BC_3_ generations for introgression lines that were then self-fertilized and germinated (BC_2_F_1_, BC_3_F_1_) to develop disomic progenies.

### Detection of introgressions using SNP markers and mcGISH

The Axiom^®^ Wheat-Relative Genotyping array was used to screen genomic DNA isolated from 195 plants of the BC_1_ to BC_4_ generations of the wheat–*Th. intermedium* hybrids. Within the array, 3414 SNPs were found to be polymorphic between *Th. intermedium* and wheat (Table S2). The sequence information for each marker, its location on the wheat genome, as well as the SNP allele in wheat and *Th. intermedium* are given in Table S3. The Affymetrix software classified the scores for each SNP into one of the six cluster patterns. However, only those classified as Poly High Resolution (PHR) and the selected Call Rate Below Threshold (CRBT) were used for linkage mapping as these are considered to be optimum quality. The highest number of SNPs was detected in homoeologous group 5 (18.5%), while homoeologous group 2 had the fewest SNPs (12.3%) (Table S2). The SNPs were analysed by JoinMap^®^ leading to the establishment of fourteen linkage groups consisting of 634 SNPs. Although *Th. intermedium* carries three chromosomes for each homoeologous group (St, J^r^ and J^vs^ genomes), SNPs could only be sorted in two types of linkage groups, one for the St genome and one for the J^r^ genome. Of the 197 lines genotyped, 187 lines showed introgressions from *Th. intermedium*. McGISH analysis of a selection of 101 progenies from the BC_2_, BC_3_ and BC_4_ generations revealed that the majority of the introgressions were monosomic additions carrying one or multiple *Th. intermedium* chromosomes and these were later used to distinguish between the linkage groups as belonging to either the St or J^r^ genome. The low number of wheat–*Th. intermedium* recombinant chromosomes thus meant that we could not produce a meaningful genetic map of the linkage groups even though the markers within each linkage group were strongly linked and detected introgressions when present. Therefore, the marker order of each linkage group was determined based on their physical position on the wheat chromosomes as obtained through BLAST analysis against the wheat reference sequence (RefSeq v1.0; International Wheat Genome Sequencing Consortium et al. 2018). Once assigned to a homoeologous group, the linkage group was then assigned to the J^r^ or St genome based on the mcGISH analysis of monosomic addition lines.

### Allocation of SNP markers to specific *Th. intermedium* chromosomes

**Fig. 2:**
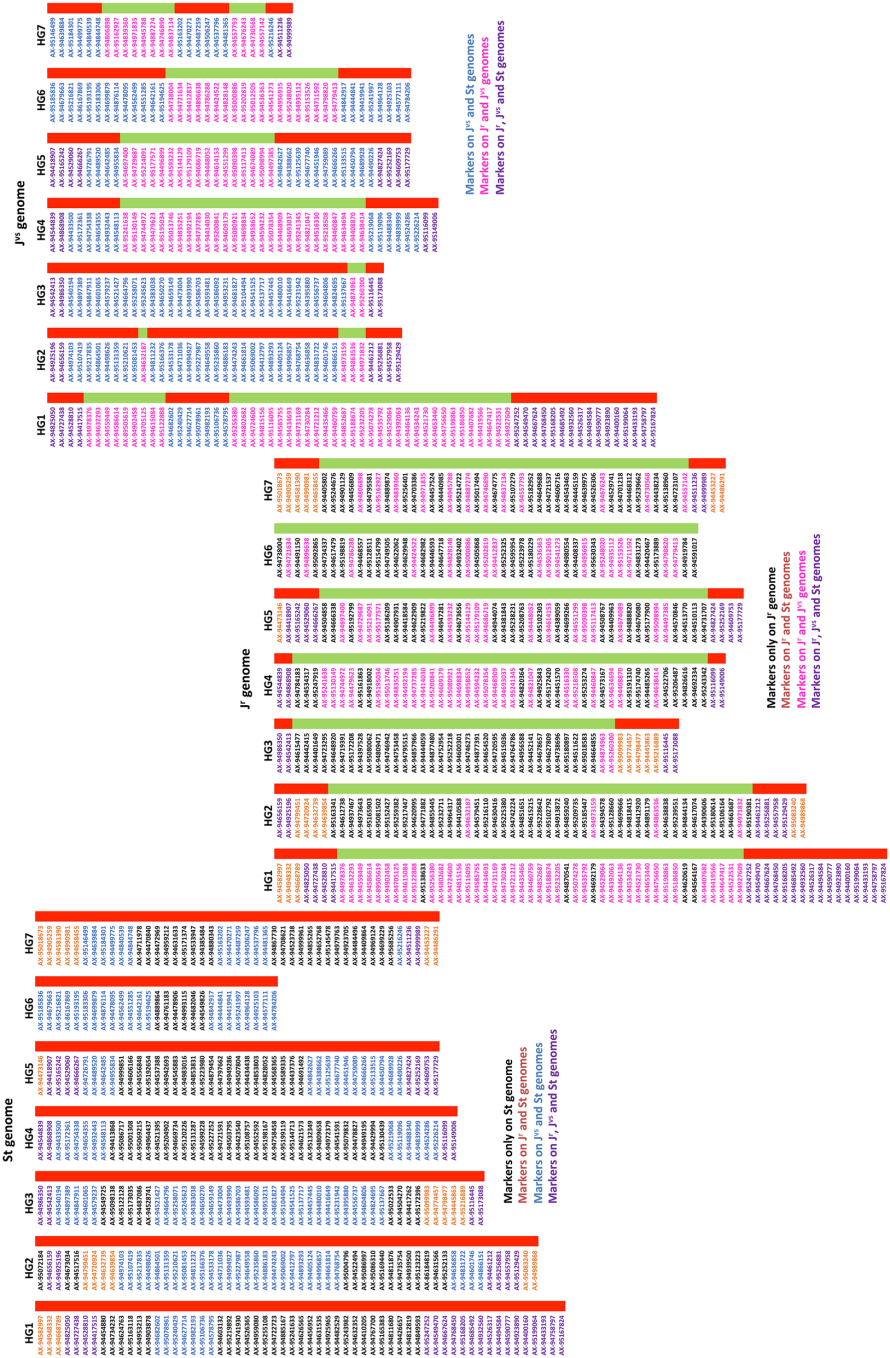
A physical map of *Th. intermedium* chromosomes containing 634 SNP markers. Physical order of markers predicted across the **a** St genome **b** Jr genome and **c** Jvs genome of *Th. intermedium*. Markers within a chromosome were organized according to cytological observations of individual chromosome’s genome constitution and ordered by their physical positions on the wheat reference sequence (RefSeq v1.0; International Wheat Genome Sequencing Consortium et al. 2018). Red areas within a chromosome show markers identified from the homoeologous group (HG) of the St genome and green areas contain markers from the J^r^ homoeologous group. Marker names in black indicate genome-specific markers. Marker names in orange represent markers present on both J^r^ and St genomes. Marker names in blue show markers present on J^vs^ and St genomes. Marker names in pink show markers present on J^r^ and J^vs^ genomes, and purple markers are present on all three (St, J^r^, J^vs^) genomes of *Th. intermedium*. The distribution and number of markers within each chromosome are also provided in Table S4 and Table 2, respectively

St genomic probe labelled the telomeric regions of the J^r^ and J^vs^ chromosomes and the centromeric regions of four different J^vs^ chromosomes (Fig. 1a–b). This raised the possibility that J^r^ and J^vs^ chromosomes would carry St genome markers at the telomeric regions and some J^vs^ chromosomes would also carry St genome markers at their centromeres. Genotyping data of the 197 plants were arranged into families according to the BC_1_, BC_2_ and BC_3_ parental lines they originated from. Within a family, genotyping data were interpreted together with the mcGISH results available for 101 plants, making it possible to assign linkage groups to individual St or J^r^ chromosomes. It was found that the 14 linkage groups as identified through JoinMap^®^ could be assigned to seven St and seven J^r^ chromosomes of *Th. Intermedium* (Fig. 2a–c, Table S4), thus making a physical SNP map for both genomes. Any lines carrying 1–3 *Th. intermedium* chromosomes or chromosome segments were used in the analysis according to their respective homoeologous groups (Table S4).

A linkage group was assigned to the St genome when all its markers were called as the allele for *Th. intermedium* in the genotyping data of an introgression line carrying a whole St chromosome as identified by mcGISH. This St linkage group was subsequently assigned to a chromosome group based on its physical position in the wheat genome, due to its homology with the wheat genome, obtained through BLAST analysis. In this way, it was possible to assign seven linkage groups to seven St chromosome groups of *Th. intermedium*. In total, there were 329 markers on the St genome map (Fig. 2a); however, only 135 markers from these seven linkage groups were specifically detecting St chromatin (Table 1) in the introgression lines as indicated by the black-coloured markers. The rest of the markers were detecting the J^r^ and/or J^vs^ genomes in addition to the St genome (markers in orange, blue and purple in Fig. 2a).

**Table 1:** Number of chromosome-specific SNP markers between *Th. intermedium* and hexaploid wheat for each homoeologous group (HG)

	Markers only on St	Markers only on J^r^	Markers on J^r^ + J^vs^	Markers on J^vs^ + St	Markers on J^r^ + St	Markers on J^r^ + J^vs^ + St	Unique markers*	All markers on St	All markers on J^r^	All markers on J^vs^
HG 1	29	5	40	7	3	20	104	59	68	67
HG 2	15	43	4	29	6	6	103	56	59	39
HG 3	10	34	2	31	5	4	86	50	45	37
HG 4	31	19	27	12	0	4	93	47	50	43
HG 5	21	26	17	15	1	8	88	45	52	40
HG 6	6	28	19	21	0	0	74	27	47	40
HG 7	23	29	12	13	7	2	86	45	50	27
Total	135	184	121	128	22	44	634	329	371	293

*Sum of markers from first six columns

A similar strategy as above was used to assign the remaining seven linkage groups to seven J^r^ chromosome groups of *Th. intermedium*. Genotyping data of the introgression lines carrying a whole J^r^ chromosome always showed the presence of all the markers from that J^r^ linkage group alongside some markers from its homoeologous St linkage group. Since mcGISH did not detect any additional translocations from the St genome in these lines, the St genome markers were considered to be potentially present as a result of the presence of St chromatin at the telomeres of the J^r^ chromosomes (Fig. 1a) and were thus physically ordered at the telomeres of the J^r^ chromosomes. This was observed for all J^r^ chromosomes except 6J^r^ that lacked the homoeologous St genome markers (Fig. 2b). The J^r^ genome physical SNP map consisted of 371 markers of which 184 were found to specifically detect the J^r^ chromosomes (Table 1). The rest of the markers were detecting the presence of St and/or J^vs^ chromosomes in addition to the J^r^ genome (markers in orange, pink and purple in Fig. 2b).

In the case of the J^vs^ genome, genotyping was guided by the mcGISH results of BC_2_ and BC_3_ lines that carried individual J^vs^ chromosomes. Due to sequence homology between the J^vs^ and J^r^ genomes (Mahelka et al. 2011), markers from the J^r^ linkage groups were found to detect J^vs^ chromosomes. Seven different SNP patterns, each consisting of a combination of St and J^r^ markers from the same homoeologous group, allowed detection of all seven J^vs^ chromosomes, validating the mcGISH results, and were subsequently formed into a physical map of the J^vs^ genome (Fig. 2c) consisting of a set of 293 markers from the St and J^r^ linkage groups. St genome markers were present at all the telomeric regions of the J^vs^ chromosomes which was also confirmed by a St-specific mcGISH signal at the telomeres of the J^vs^ chromosomes (Fig. 1b). St genome markers were also found to be present at the centromeric regions of chromosomes 1J^vs^, 2J^vs^, 3J^vs^and 7J^vs^ (blue markers in Fig. 2c) which validates the mcGISH results that indicated four J^vs^ chromosome pairs carrying St signals at their centromeres (Fig. 1b). It was expected that majority of the markers that detect the presence of the J^vs^ chromosomes would be from the J^r^ genome (markers in pink in Fig. 2c) since the St genome signal is found in small regions of the J^vs^ chromosomes, either at the telomeres or the centromeres. However, it was noted that chromosomes 2J^vs^ and 3J^vs^ were predominantly detected by St genome markers (Fig. 2c).

**Fig. 3:**
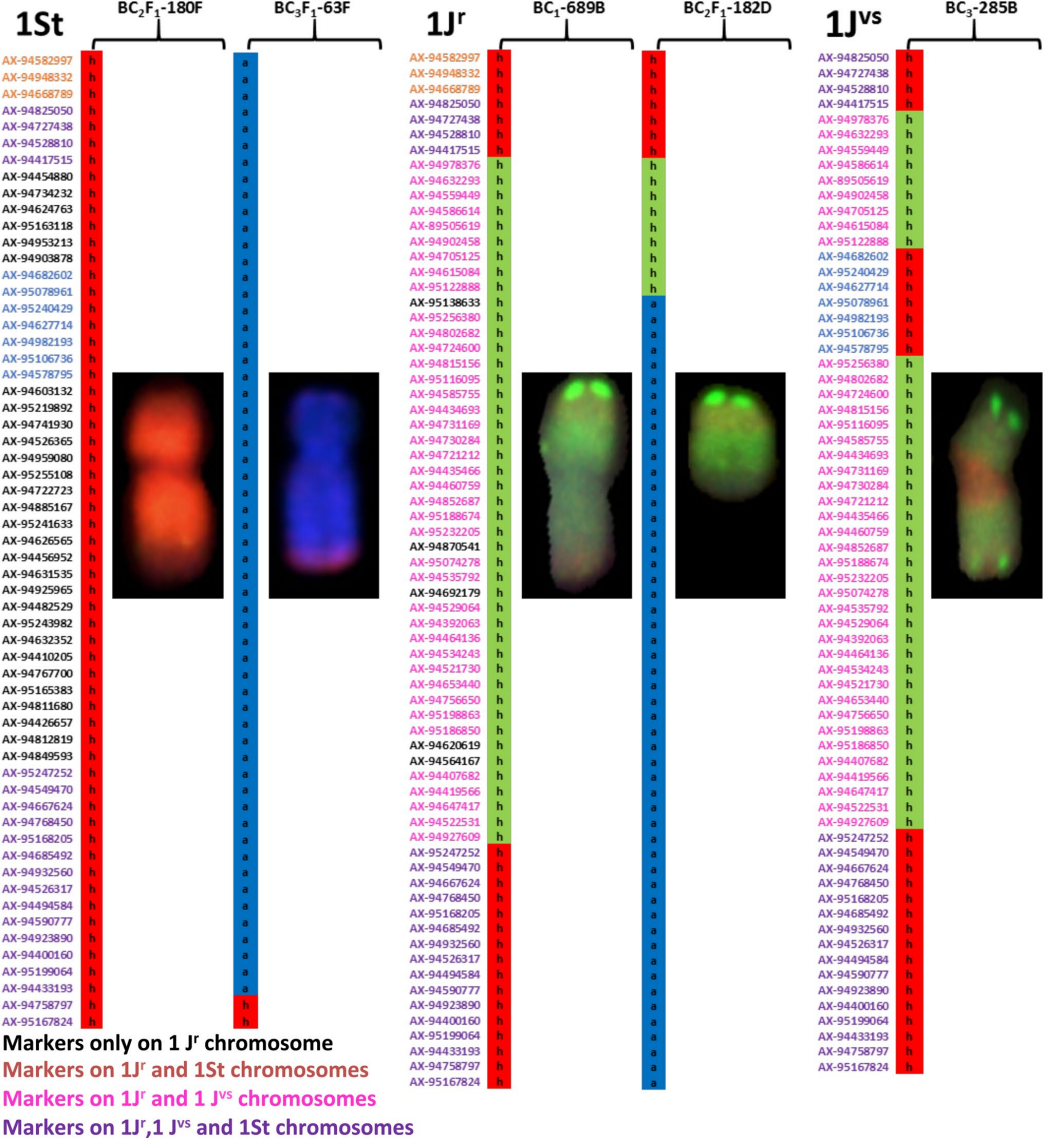
Homoeologous group 1-specific SNP markers showing the 1St, 1J^r^ and 1J^vs^ chromosomes of *Th. intermedium*. The chromosome inserts show the corresponding wheatgrass chromosomes and chromosome segments identified by mcGISH. The mcGISH images show metaphase chromosomes probed with labelled genomic DNA of *Th. bessarabicum* (green) and *Pseudoroegneria spicata* (red). Presence of a heterozygous call for an SNP marker, represented in red and depicted as ‘h’ (for heterozygous), indicated the presence of *Th. intermedium* St genome in a wheat background, and the green heterozygous markers indicated the presence of *Th. intermedium* J^r^ genome. The homozygous call, represented in blue and depicted as ‘a’ (for parent ‘a’, i.e. wheat), indicated the absence of *Th. intermedium* in wheat at that marker. Marker names indicated in pink are markers common between J^r^ and J^vs^. Marker names in blue are common between St and J^vs^. Marker names in orange are common between J^r^ and St, and marker names in purple are common to all three genomes. Marker names shown in black letters are unique to that genome

Figure 3 shows an example of how the above assignment of markers to homoeologous group 1 of *Th. intermedium* chromosomes was validated by mcGISH observations. In line BC_2_F_1_-180F, carrying a whole St chromosome, all 59 markers from the chromosome 1St were present. In contrast, line BC_3_F_1_-63F carrying a chromosome with a small segment of the St genome at the telomere of its long arm showed the presence of only two markers at the distal end of chromosome 1St, indicating the presence of a wheat–*Th. intermedium* recombinant chromosome from linkage group 1St. Line BC_1_-689B showed the presence of a J^r^ chromosome (probed with *Th. bessarabicum* gDNA showing a green signal through the length of the chromosome and St genome signal as red at both telomeres) which was validated by the presence of all 68 markers assigned to chromosome 1J^r^ (which consisted of a mix of markers from the 1J^r^ and 1St linkage groups). In contrast, line BC_2_F_1_-182D carrying a telosome from the J^r^ genome only showed the presence of the top 16 markers assigned to chromosome 1J^r^, indicating the presence of its short arm in this line. In line BC_3_-285B, mcGISH results showed the presence of a J^vs^ chromosome as indicated by the presence of the St genome signal (red) at its centromere in addition to both telomeres. This line showed the presence of all 67 markers that were assigned to chromosome 1J^vs^ (which consisted of a mix of markers from the 1J^r^ and 1St linkage groups), thereby validating the assignment of these markers to detect chromosome 1J^vs^. Detailed genotyping of all introgressions from *Th. intermedium* in lines in Fig. 3 and in additional lines (used to validate the assignment of markers to the chromosomes) is given in Table S4.

A total of 634 markers were identified of which 135 were St genome-specific and 184 J^r^ genome-specific; thus, almost half of the markers were present on multiple genomes of the same homoeologous group (Table 1). Of the total, 44 markers from the St genome detected chromosomes across the St, J^r^ and J^vs^ genomes (purple markers in Fig. 2a–c; Table 1). The allocated markers are codominant and were proven useful to trace the *Th. intermedium* chromosomes and chromosome segments in a wheat background.

### Identification of new recombinant lines by SNP genotyping, GISH and FISH

The newly developed *Th. intermedium* chromosome-specific marker set was found to be effective in high-throughput identification of alien chromosomes/segments in a wheat background. A subset of genotyped lines is presented in Table 2, showing which *Th. intermedium* chromosome and/or chromosome segment is present, as indicated by the SNP genotyping and validated by mcGISH analysis. The SNP map clearly pinpointed the addition of complete *Th. intermedium* chromosomes (St, J^r^ or J^vs^) to the wheat genome; however, identification of the recombinant chromosomes proved more challenging as they required a combination of SNP genotyping, GISH and FISH to fully characterize the recombinant chromosome. On the other hand, the SNP genotyping reduced the number of plants subject to laborious in situ hybridization and allowed clear identification of recombinant lines in a time-effective manner.

**Table 2:** Wheat/*Th. intermedium* monosomic addition lines as detected by SNP genotyping and multicolour genomic in situ hybridization (mcGISH). The identification of the *Th. intermedium* chromosomes is based on the SNP marker positions in homoeologous groups of wheat and mcGISH data

Accession number of plants	Wheat/*Th. intermedium* introgression lines	SNP characterization of *Th. intermedium* chromosomes (homoeologous group/St or J^r^ genome-specific SNP’s)	mcGISH (number of St, J^r^ and J^vs^ chromosomes)
BC_2_F_1_-180F	1St + telosomic 2J^vs^ − S	1/St, 2/St	1 St + 1 telosomic J^vs^
BC_2_-689B	1J^r^ + 2J^vs^ − L centric fusion	1/J^r^, 1/St, 2/St, 2/J^r^	1 J^r^ + 1 J^vs^ centric fusion
BC_2_F_1_-182D	telosomic 1J^r^ − S	1/J^r^, 1/St	1 telosomic J^r^
BC_3_-332B	2St	2/St	n.a.
BC_3_-339A	2J^r^	2/J^r^, 2/St	1 J^r^
BC_3_-345B	2J^vs^	2/J^r^, 2/St	1 J^vs^
BC_3_-337A	3J^r^	3/J^r,^ 3/St	1 J^r^
BC_3_-301A	4J^r^ + 7J^vs^	4/J^r^, 4/St, 7/J^r^, 7/St	1 J^r^ + 1 J^vs^
BC_3_-346A	5St	5/St	n.a.
BC_3_-340B	5J^r^ + 7St	5/J^r^, 5/St, 7/St	1 J^r^ + 1 St
BC_3_-335B	6St-6J^vs^ (translocation)	6/St, 6/J^r^	1 St-J^vs^
BC_3_-344A	7St	7/St	1 St
BC_3_F_1_-62A	7J^r^	7/J^r^, 7/St	1 J^r^

**Fig. 4:**
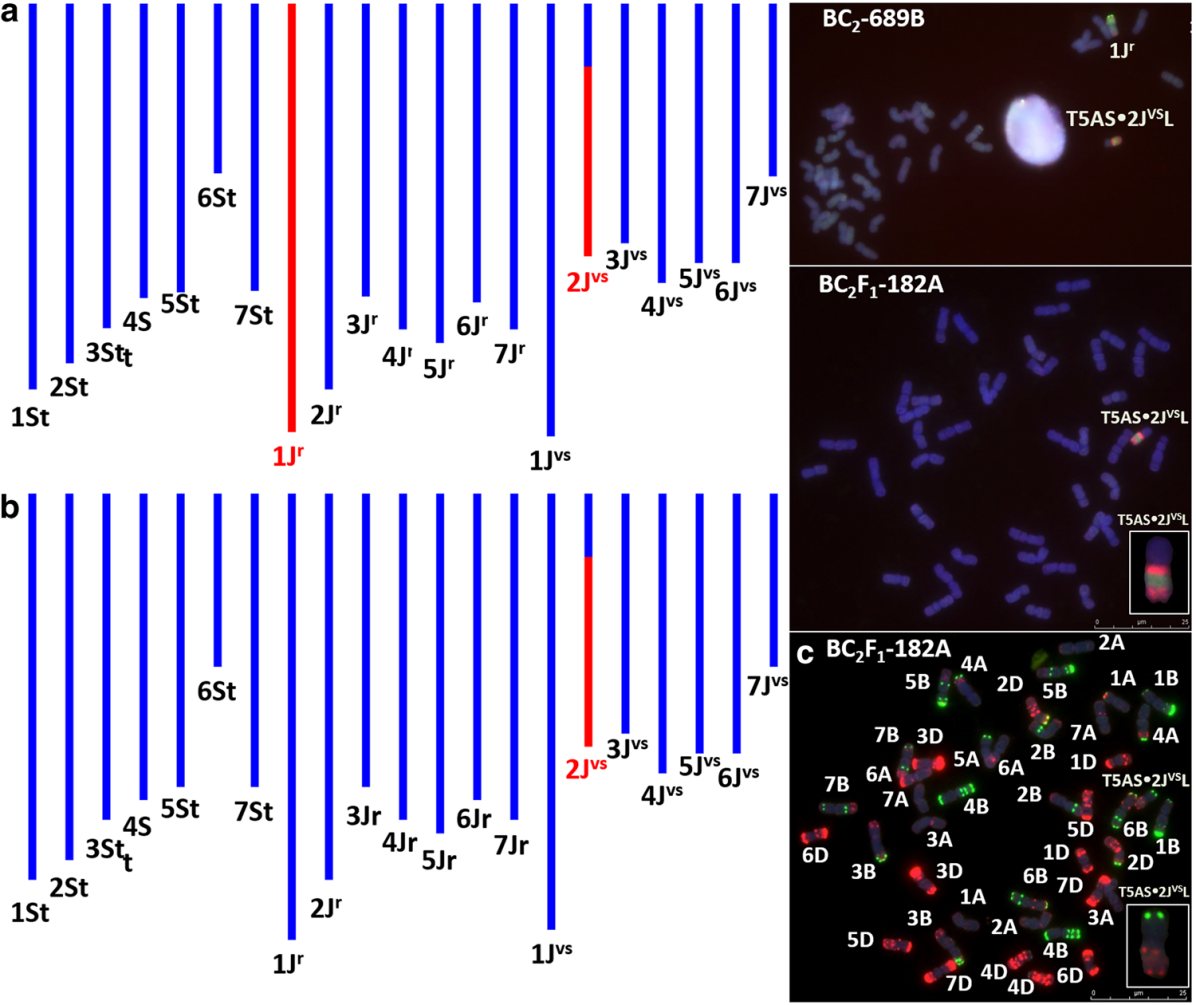
SNP characterization of wheat/*Th. intermedium* introgression lines together with in situ hybridization results. In the SNP characterization, red colour is used to show the presence of a *Th. intermedium* introgression, while blue colour represents the absence of *Th. intermedium*-specific alleles in the GGT bar diagram. The mcGISH image shows a metaphase spread of BC_2_-689B (**a**) and BC_2_F_1_-182A (**b**) (progeny of BC2-689B) probed with labelled genomic DNA of *Th. bessarabicum* (green) and *Pseudoroegneria spicata* (red). The BC_2_-689B is a monosomic 1J^r^ addition line containing the T5AS·2J^vs^L recombinant chromosome. The long arm of the 2J^vs^ chromosome shows the J^vs^ genome-specific hybridization pattern of the *Pseudoroegneria spicata* probe on the centromeric and telomeric regions. The 1J^r^ chromosome is labelled with *Th. bessarabicum* genomic DNA (green) and shows dispersed red signal (*Pseudoroegneria spicata*) at the telomeric regions. **c** FISH on mitotic chromosomes of the wheat/*Th. intermedium* BC_2_F_1_-182A (T5AS·2J^vs^L) recombinant line with probes for DNA repeats: Afa family (red), pSc119.2 (green)

After identification of recombinant lines, it was possible to track the *Th. intermedium* segments in their progenies with the help of SNP genotyping and mcGISH, as shown in Fig. 4a–c. In line BC_2_-689B, mcGISH and marker data confirmed the presence of chromosome 1J^r^ and the long arm of chromosome 2J^vs^ recombined with wheat (Fig. 4a). Marker data on the subsequent generation of the plant allowed to select a putative wheat–*Thinopyrum* recombinant line, BC_2_F_1_-182A, showing the loss of chromosome 1J^r^ and retention of the 2J^vs^L chromosome arm validated by the mcGISH analysis (Fig. 4b). Subsequently, the recombinant chromosome was identified as a T5AS.2J^vs^L wheat/*Th. intermedium* centric fusion by FISH analysis (Fig. 4c).

**Fig. 5:**
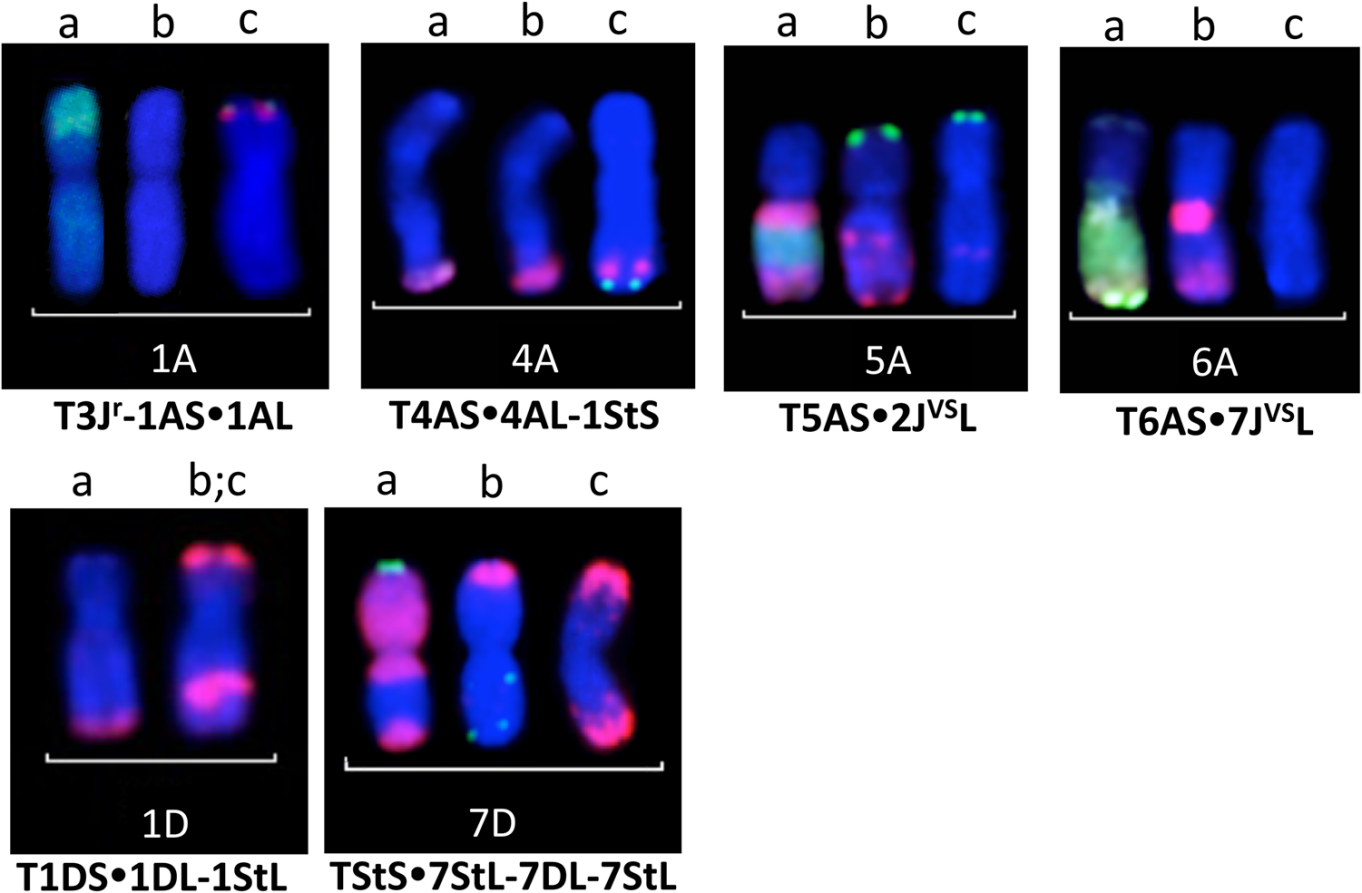
Sequential multicolour GISH and FISH analysis of the recombinant chromosomes detected in the wheat/*Th. intermedium* recombinant lines presented together with the FISH pattern of the wheat chromosomes. **a** Visualization of the alien chromatin with GISH by using labelled genomic DNA of *Thinopyrum bessarabicum* (green) and *Pseudoroegneria spicata* (red); **b** FISH pattern of the recombinant chromosomes with Afa family (red) and pSc119.2 (green) repetitive DNA probes; **c** FISH pattern of the corresponding ‘Paragon’ wheat chromosomes with Afa family (red) and pSc119.2 (green) repetitive DNA probes. Chromosomes are counterstained with DAPI (blue)

SNP genotyping together with GISH and FISH identified six recombinant lines in total, as shown in Fig. 5. There were three telomeric translocations, two of which were non-homoeologous (T3J^r^S-1AS.1AL and T4AS.4AL-1StS) and one homoeologous (T1DS.1DL-1StL). Subsequently, two non-homologous centric fusions were also identified (T5AS.2J^vs^L and T6AS.7J^vs^L). Finally, the largest wheat/*Th. intermedium* translocation chromosome included chromosome 7D of wheat and 7St of *Th. intermedium* (T7StS.7StL-7DL-7StL). All *Th. intermedium* telosomics and recombinant chromosomes identified during the crossing programme are concluded in Table S4. Recombinant chromosomes identified by GISH and FISH were also useful in validating the SNP map. Moreover, the new wheat/*Th. intermedium* introgression lines can be used as a valuable gene tool in future wheat improvement programmes. Introgression lines will be bulked and made available via the Nottingham/BBSRC Wheat Research Centre website at http://www.nottingham.ac.uk/wisp.

### Syntenic relationship between wheat and *Th. intermedium*

The sequences from a total of 634 markers allocated on the *Th. intermedium* chromosomes were used in BLAST analysis against the wheat Chinese Spring genome assembly (Refseqv1, International Wheat Genome Sequencing Consortium et al. 2018). The BLAST hits from each of the three wheat genomes, if the identity percentage was greater than 95% (Table S4), were noted wherever possible along with the best BLAST hit. 72.6% of the markers had a significant BLAST hit on all three genomes of wheat. The BLAST analysis showed that 86.4%, 91.9% and 92.7% had a significant BLAST on the A, B and D genomes of wheat, respectively. Of these BLAST hits, 35.6%, 40.7% and 50.8% of the markers had an overall top hit on the A, B and D genomes of wheat, respectively, indicating that the D genome of wheat has the closest synteny with the *Th. intermedium* genomes.

**Fig. 6:**
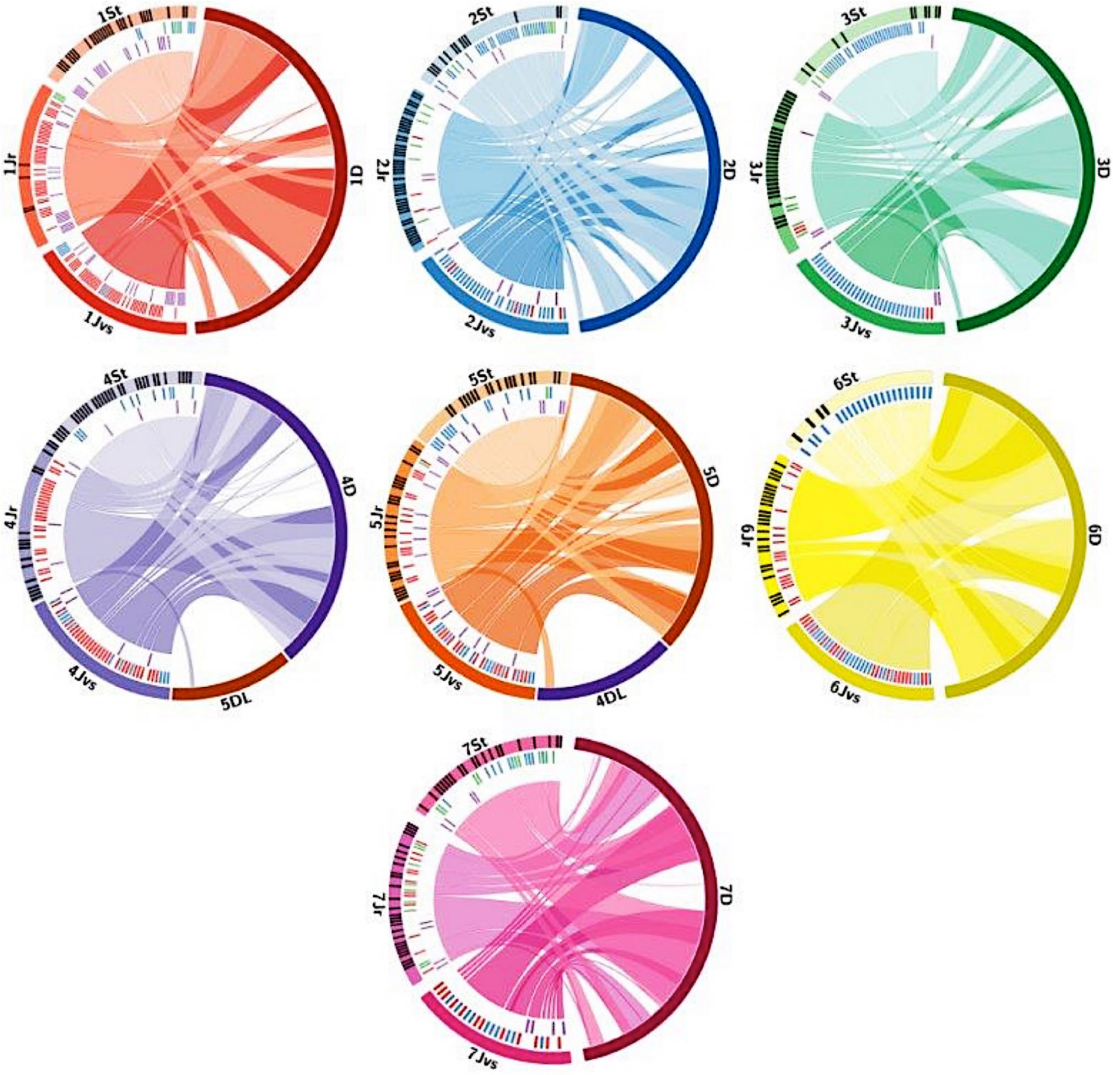
Comparative analysis of synteny between the *Th. intermedium* subgenomes and the D genome of hexaploid wheat. The marker order on the *Th. intermedium* chromosomes is as presented in the physical map. Markers indicated by a black line within the chromosome ideograms are unique to the individual genomes. Markers showed by a red line are common between J^r^ and J^vs^, markers represented by blue lines are common between St and J^vs^, and markers indicated by green lines are common between J^r^ and St genomes, and purple lines show markers common to all three genomes. Large ribbons represent syntenic relationships between *Th. intermedium* subgenomes and their homoeologous chromosomes in the D genome of wheat (RefSeq v1.0; International Wheat Genome Sequencing Consortium et al. 2018)

Figure 6 uses large ‘ribbons’ to show homoeology between the 21 chromosomes of *Th. intermedium* and the D genome of wheat. Circos plots indicate a significant syntenic relationship between the 21 chromosomes of *Th. intermedium* and their homoeologues from the D genome of wheat. Markers within each of the *Th. intermedium* subgenomes are indicated with different coloured lines within or radially next to the ideogram, representing the subgenome chromosome in each of the seven homoeologous groups. The difference in the coloured lines represents the distribution of the markers across the subgenomes as described in Fig. 2a–c. The subtelomeric region on the long arm of 4 J^r^ carried a few markers, unique to the J^r^ genome, which mapped to chromosome 4A of wheat but were located on the 5th homoeologous group within the B and D genomes. Similarly, some 5J^r^ markers were mapped to the 5A, 4B and 4D chromosomes, indicating that *Th. intermedium* 4J^r^ and 5J^r^ chromosomes carry the 4/5 translocation such as the 4A/5A translocation observed in wheat (Liu et al. 1992; Devos et al. 1995). The 4 and 5 chromosomes of St and J^vs^ genomes did not show any inter-chromosomal translocation (Fig. 6, Table S4).

## Discussion

*Thinopyrum intermedium* has been historically used as a source of desirable traits in wheat breeding programmes. In particular, it provides superior resistance against various fungal and viral diseases of wheat (Li and Wang 2009); therefore, understanding its genomic composition is of great interest. In the present study, we provide a new valuable tool to accurately trace *Th. intermedium* chromatin transferred to wheat useful for wheat improvement programmes. Despite the long-standing scientific interest on transferring and analysing *Th. intermedium* chromatin in bread wheat, our approach is the first allowing the accurate and high-throughput identification of the individual *Th*. *intermedium* chromosomes or small chromosome segments in wheat introgression lines. In addition to SNP marker development, we produced and identified a range of new wheat genetic material consisting of different chromosomes or chromosome segments originating from *Th. intermedium* and potentially carrying agronomically advantageous traits. The developed technique and introgression lines are readily applicable in wheat improvement programmes to effectively transfer valuable characteristics from *Thinopyrum* species into wheat.

While numerous studies aimed to specify the allohexaploid genome composition of *Th. intermedium* itself, its complex nature made it difficult to accurately define the individual subgenomes, a task further encumbered by the polymorphism observed within different accessions and eventual intergenomic rearrangements (Liu and Wang 1993; Wang et al. 2015). Early genome evolution studies used in situ hybridization with St, J and E genomic DNA probes and proposed the presence of three distinguishable chromosome sets designated as St, J and J^S^ genomes. However, only 6–11 chromosomes were found to belong to the J^S^ genome, which were identified by the presence of the St signal within the pericentromeres (Liu and Wang 1993; Chen et al. 1998). Mahelka et al. (2011) demonstrated that V genomic probe originating from *Dasypyrum villosum* labels 14 chromosomes (7 pairs) of *Th. intermedium,* nine of which showed St genomic signal within the pericentromeric regions. In the present study, SNP genotyping supported by multicolour genomic in situ hybridization clarified and provided a more detailed insight into the genetic make-up of *Th. intermedium*.

We distinguished the genomes of *Thinopyrum intermedium* (StStJ^r^J^r^J^vs^J^vs^, 2*n* = 6*x* = 42) in two different accessions by sequential multicolour GISH and revealed 14 St genome chromosomes by St genomic probe (*Pseudoroegneria spicata*), 14 J^r^ genome chromosomes by J^b^ genomic probe (*Thinopyrum bessarabicum*) and 14 J^vs^ chromosomes distinguished by V genomic probe (*Dasypyrum villosum*) (Fig. 1). Eight (four pairs) of the 14 J^vs^ chromosomes showed St genomic signal on the centromeric regions, which were subsequently identified as 1J^vs^, 2J^vs^, 3J^vs^ and 7J^vs^ chromosomes by SNP genotyping (Fig. 2c). However, it is yet to be elucidated whether all *Th. intermedium* accessions carry centromeric St sequences on the 1J^vs^, 2J^vs^, 3J^vs^ and 7J^vs^ or are these centromeric rearrangements present on other J^vs^ chromosomes as well.

A range of chromosome engineering techniques have been applied previously to produce wheat–wild relative introgressions lines. Here, we induced homoeologous chromosome pairing in wheat–*Th intermedium* introgression lines by using a *ph1* mutant line as the female parent, similar to that described by King et al. (2017). Amphihaploid F_1_ hybrids between hexaploid wheat (*ph1/ph1*) and *Th. intermedium* were created, and homoeologous recombination was expected to occur at meiosis in the F_1_ hybrids. Further backcrosses with hexaploid wheat (*Ph1/Ph1*) have been used to eliminate the unpaired *Th. intermedium* chromosomes and to obtain recombinant lines carrying only a segment of the *Th. intermedium* genome. Since the interspecific F_1_ hybrids were haploid for the A, B, D and St, J^r^, J^vs^ genomes, their fertility was predicted to be low and this was found to be the case (Table S1). However, their fertility (30.5%) was higher than that previously observed in those between wheat and *Ae. speltoides* (29%), wheat–*Am. muticum* (16.2%) and wheat–*Th. bessarabicum* (1.4%) (King et al. 2017, 2018; Grewal et al. 2018). The fertility of BC_1_ generation was 62.8%, while BC_3_ plants had a fertility as high as 98.5%, most likely because the majority of the unpaired introgressed *Th. intermedium* chromosomes had been eliminated. As a result of repeated backcrossing, wild relative chromosomes are randomly eliminated in subsequent generations leading to numerous combinations of the wild relative chromosomes in the progenies. Nine different monosomic addition lines were selected, and four lines carried only two different *Th. intermedium* chromosomes or chromosome arms (Table 1). Among the 51 BC_3_ plants, ten did not contain introgression chromosomes or translocations while such a complete elimination of the *Th. intermedium* genomes was not observed in earlier generations. Random elimination of the wheatgrass chromosomes suggested that *Th. intermedium* does not carry highly effective gametocidal genes in contrast to *Aegilops cylindrica, Ae. sharonensis* or *Ae. speltoides* (Endo 1988; King et al. 1991, 2018).

The present study genotyped various wheat–*Th. intermedium* introgression lines to map 634 SNP markers specific to individual *Th. intermedium* chromosomes (Table S2) and validated the results using mcGISH. Using available sequence information from wheat (International Wheat Genome Sequencing Consortium et al. 2018), we assigned the markers to their known chromosome locations in the wheat genome and generated an integrated physical map including 21 chromosomes of *Th. intermedium* (Fig. 2a–c). Validation of introgressions identified by SNP analysis was carried out by mcGISH analysis, and the number of wheat/*Th. intermedium* introgressions detected by the SNP analysis corresponded to the number of introgressions detected by the mcGISH (Table 1, Figs. 3 and 4). The SNP marker set developed in this study will dramatically increase the density of the *Th. intermedium* chromosome-specific SNP markers and hence will be very helpful in identifying wild relative chromosomes or recombinant chromosomes in a wheat background. Recently, the most detailed genetic map of *Th. intermedium* was published by Kantarski et al. (2017) using genotyping-by-sequencing. They used wheatgrass full-sib mapping populations and self-derived family for marker development, and thus, the markers cannot be used directly to screen wheat/*Th. intermedium* hybrid lines without knowing the degree of polymorphism for these with/within wheat.

The low fertility of the F_1_ hybrids resulted in the generation of only 33 BC_1_ seeds that grew to maturity and set seed. As intergenomic recombination did not occur in later generations, the total number of introgressions that could be generated was limited to the 33 female F_1_ gametes, giving rise to these 33 BC_1_ plants. Elimination or transmission of *Th. intermedium* recombinant chromosomes in the BC_2_ and BC_3_ generation was traced with the help of the SNP markers (Fig. 4). By using the SNP map and mcGISH, we detected 12 different chromosome arms in the progenies as telocentrics or centric fusions (Table S4). Two centric fusions were analysed by mcGISH and FISH, and the translocation chromosomes were identified as T5AS.2J^vs^L and T6AS.7J^vs^L (Figs. 4 and 5). These centric fusions and telosomic lines are most likely misdivision products and not crossover-derived recombinants, and chromosome engineering techniques are needed to shorten the alien chromosome segments before directly applying in wheat breeding programmes.

SNP genotyping identified nine different wheat/*Th. intermedium* recombinant chromosomes where the *Th. intermedium* segment was either shorter or longer than one chromosome arm (Table S4). Four of these recombinant chromosomes were also identified by mcGISH and FISH that showed that only two lines (T1DS.1DL-1StL and T7StS.7StL-7DL-7StL) carried homoeologous recombinant chromosomes (Fig. 5). The T7StS.7StL-7DL-7StL recombinant chromosome was developed in two steps. The BC_2_-688 plant carried the T7StS.7StL-7DL translocation and a 7St wheatgrass chromosome. The T7StS.7StL-7DL-7StL recombinant line was found in the BC_2_F_1_ generation. That was the only case resulting in a new recombinant chromosome found during self-fertilization and backcrossing. The low number of wheat/*Th. intermedium* homoeologous recombinants indicated inhibition of pairing between the wheat and the *Th. intermedium* chromosomes. In contrast, Patokar et al. 2016 and Grewal et al. 2018 developed a number of wheat/*Th. bessarabicum* homoeologous recombinant lines where recombinations of *Th. bessarabicum* chromosomes involved all three (A, B, D) genomes of wheat. Although the diploid J^b^ genome of *Th. bessarabicum* is closely related to the J^r^ and J^vs^ genomes of *Th. intermedium*, wheat/wheat homoeologous chromosome pairing and autosyndetic pairing between intermediate wheatgrass chromosomes appears to be preferred in wheat/*Th. intermedium* hybrids over wheat–wild relative pairing. Autosyndetic pairing of homoeologous *Th. intermedium* chromosomes in hybrids with *Triticum* has been extensively studied, and low levels of pairing have been consistently detected between the wheat and *Thinopyrum* chromosomes. It has been proposed that in wheat/*Th. intermedium* hybrids carrying a dominant copy of the *Ph1* gene, a promoter gene originating from *Th. intermedium* facilitates autosyndetic pairing (Cai and Jones 1997; Cai et al. 2001; Chen et al. 2001). Our study used a *ph1* mutant wheat line as the female crossing partner and still did not detect a large number of wheat/*Th. intermedium* homoeologous recombinants, supporting the theory that a promoter gene or chromosome recognition mechanism, similar to the wheat *Ph*-pairing system, is present in the polyploid *Th. intermedium* genome and appears to function effectively in the *ph1* mutant background as well.

Comparative analysis between *Th. intermedium* and wheat chromosomes showed a macro-synteny between the 21 chromosomes of *Th. intermedium* and their homoeologues from the A, B and D genomes of wheat. Most of the wheatgrass chromosome-specific markers had a significant BLAST hit on all three genomes of wheat and with most having top BLAST hits on the D genome of wheat, indicating that *Th. intermedium* was more closely related to the D genome of wheat. Previous work by Liu et al. (2007) has also showed that other *Thinopyrum* genomes such as the E genome of diploid *Thinopyrum elongatum* are more closely related to the D genome of wheat. Thus, Fig. 6 is constructed using the D genome of wheat and shows significant syntenic relationships between the chromosomes of *Th. intermedium* and their homoeologues from the D genome of wheat. The subtelomeric region on the long arm of 4 J^r^ links to the distal regions of the 5DL, and the markers from the distal end of 5J^r^L links to the distal regions of the long arm of 4D. This indicates a reciprocal translocation between the long arms of chromosomes 4J^r^ and 5J^r^ which confirms previous reports by King et al. (1994) and Grewal et al. (2018), involving a 4/5 translocation within the *Th. bessarabicum* J^b^ genome. This emphasizes that *Th. intermedium* J^r^ genome is distinct from the St and J^vs^ genomes that are missing the 4/5 reciprocal translocation.

In this study, we generated and validated a new chromosome-specific, easy-to-use SNP marker set that can be used to characterize and identify the *Th. intermedium* chromosomes or chromosome segments transferred into wheat–*Th. intermedium* introgression lines. Additionally, we used cytogenetic methods to clarify the genome constitution of the parental *Th. intermedium* accessions. These findings have provided a more complex overview of the intermediate wheatgrass genome at three conceptual levels: genome, chromosome and DNA.

### Author contribution statement

AC, SG, CY, SHE, DS, SA, IPK and JK carried out the crossing programme. AC performed the in situ hybridization experiments. SHE, DS, SA, AC and CY prepared the samples for genotyping, and AJB ran the samples on the array. SG and AC analysed the genotyping data and constructed the physical map. SG, AC and PW performed the comparative genome studies. AC, SG, IPK and JK conceived and designed the experiments. AC and SG wrote the manuscript with assistance from JK and IPK. All authors read and approved the final manuscript.

## Electronic supplementary material

Below is the link to the electronic supplementary material.

**Table S1.** Number of seeds produced and germinated in relation to the number of crosses carried out for each generation of the introgression programme for *Th. intermedium* into wheat (DOCX 17 kb)

**Table S2.** Number of polymorphic Poly High Resolution (PHR) and Call Rate Below Threshold (CRBT) SNPs between *Th. intermedium* and hexaploid wheat, for each homoeologous group (HG), in total on the 35K Axiom^®^ Wheat-Relative Genotyping array and those used in the linkage analysis (DOCX 16 kb)

**Table S3.** The sequence information of polymorphic SNPs between *Th. intermedium* and wheat (XLSX 482 kb)

**Table S4.** SNP characterization of wheat/*Th. intermedium* introgression lines together with *in situ* hybridization results and SNPs positions on IWGSC wheat survey sequence (RefSeq v1.0; International Wheat Genome Sequencing Consortium et al. 2018). (XLSX 171 kb)
