# Supplementary material for: Development and validation of an exome-based SNP marker set for identification of the St, Jr and Jvs genomes of Thinopyrym intermedium in a wheat background
Source: Theor Appl Genet. 2019 Feb 14;132(5):1555–70. doi: 10.1007/s00122-019-03300-9 (PMC6476854; doi:10.1007/s00122-019-03300-9)
Supplement: Supplementary file 1 — Table S1. Number of seeds produced and germinated in relation to the number of crosses carried out for each generation of the introgression programme for Th. intermedium into wheat (DOCX 17 kb) [file 122_2019_3300_MOESM1_ESM.docx]

**Table S1.** Number of seeds produced and germinated in relation to the number of crosses carried out for each generation of the introgression programme for *Th. intermedium* into wheat.

|  | **F_1_** | **BC_1_** | **BC_2_** | **BC_3_** | **BC_4_** | **BC_2_F_1_** | **BC_3_F_1_** | **Total** |
| --- | --- | --- | --- | --- | --- | --- | --- | --- |
| **Number of plants were genotyped** | 2 | 36 | 72 | 51 | 5 | 23 | 9 | 197 |
| **Number of seed sown** | 104 | 46 | 101 | 75 | 5 | 25 | 9 | 365 |
| **Number of seed that germinated**  **(%)** | 33  (31.7) | 40  (87.0) | 74  (73.3) | 60  (80.0) | 5  (100) | 23  (92.0) | 9  (100) | 244  (66.8) |
| **Number of plants setting seed**  **(%)** | 29  (87.9) | 33  (82.5) | 65  (87.8) | 51  (85.0) | 4  (80.0) | 20  (87.0) | 8  (88.9) | 210  (86.0) |
| **Number of seed/total number of crosses**  **(Average number of seed set per crossed ear)** | 51/131  (0.4) | 687/239  (2.9) | 787/237  (3.3) | 1757/136  (12.9) | 0/0  (-) | 171/25  (7.0) | 0/0  (-) | 3282/768  (4.3) |
| **Number of crosses producing seed**  **(%)** | 40  (30.5) | 150  (62.8) | 160  (67.5) | 134  (98.5) | - | 21  (84.0) | - | 505  (65.8) |
| **Number of self-fertilised seed produced** | 0 | 900 | 804 | 1535 | 105 | 288 | 264 | 3687 |
