# Supplementary material for: Development and validation of an exome-based SNP marker set for identification of the St, Jr and Jvs genomes of Thinopyrym intermedium in a wheat background
Source: Theor Appl Genet. 2019 Feb 14;132(5):1555–70. doi: 10.1007/s00122-019-03300-9 (PMC6476854; doi:10.1007/s00122-019-03300-9)
Supplement: Supplementary file 2 — Table S2. Number of polymorphic Poly High Resolution (PHR) and Call Rate Below Threshold (CRBT) SNPs between Th. intermedium and hexaploid wheat, for each homoeologous group (HG), in total on the 35K Axiom® Wheat-Relative Genotyping array and those used in the linkage analysis (DOCX 16 kb) [file 122_2019_3300_MOESM2_ESM.docx]

**Table S2.**Number of polymorphic Poly High Resolution (PHR) and Call Rate Below Threshold (CRBT) SNPs between *Th. intermedium* and hexaploid wheat in total on the 35K Axiom® Wheat Relative Genotyping array and those used in the linkage analysis.

|  | **HG 1** | **HG 2** | **HG 3** | **HG 4** | **HG 5** | **HG 6** | **HG 7** | **Total** |
| --- | --- | --- | --- | --- | --- | --- | --- | --- |
| **PHR**  **(% of total)** | 293  (13.6) | 281  (13.0) | 262  (12.1) | 301  (13.9) | 393  (18.2) | 303  (14.0) | 329  (15.2) | 2162 |
| **CRBT***  **(% of total)** | 221  (17.7) | 140  (11.2) | 180  (14.4) | 182  (14.5) | 240  (19.2) | 148  (11.8) | 141  (11.3) | 1252 |
| ***PHR+CRBT****  ***(% of total)*** | **514**  **(15.0)** | **421**  **(12.3)** | **442**  **(12.9)** | **483**  **(14.1)** | **633**  **(18.5)** | **451**  **(13.2)** | **470**  **(13.8)** | **3414** |
| **Linked Markers**  **(% of total)** | **104**  **(16.4)** | **103**  **(16.2)** | **86**  **(13.6)** | **93**  **(14.7)** | **88**  **(13.9)** | **74**  **(11.7)** | **86**  **(13.6)** | **634** |

*CRBT markers with greater than 6% missing data were removed
